# Editorial

**Published:** 1842-03-05

**Authors:** 


### THE MEDICAL EXAMINER.

### PHILADELPHIA, MARCH 5, 1842.

*The Cortlandville Case of Mal-Practice.*—This affair has been the subject of some recent proceedings of the Medical Society of Cortland county, New York, which we give below. A detailed notice of the facts out of which this dispute has grown, will be found in the 45th number of our last volume; but, for the benefit of our readers of the new series, we repeat the more important points of the case.

On the fourth of July, 1839, the plaintiff in the prosecution for malpractice, suffered a compound fracture of both bones of his leg, the tibia two inches, and the fibula four inches above the ankle joint. Dr. A. B. Shipman was called to see him. Finding no important vessel or nerve injured, he reduced and adjusted the fracture, closed the wound, and dressed the limb with the Scultetus bandage and short splints. The next day, the man was removed to the alms-house, and passed from the care of Dr. S. On the ninth day after, Dr. Shipman was invited to witness the amputation of the limb, and, upon attending, found several physicians in consultation upon the case. At this time, the limb lay over the double-inclined plane, the tibia protruding two inches, was dry, dark, and necrosed. "The foot was turned off," (everted,) "and the leg distorted" with muscular contraction and shortening, swollen and inflamed nearly to the knee. The wound gaped, but without loss of substance, was healing at the upper extremity, "and the granulations covered a portion of the bone. The patient suffered much pain, yet was free from fever; appetite, strength, and pulse good, tongue clean, and bowels regular." Upon consultation, there was a division of opinion. Drs. Goodyear and Hyde, with others, were for immediate amputation. Dr. Shipman, supported by some, opposed it. Nothing was done.

On the nineteenth day, the patient was permitted to place himself under the charge of Dr. Shipman. He found him in much the same condition—the limb rather more distorted—the bone still protruding. The heel, very sore from pressure on the plane, "had sloughed to the bone; the same dressing was upon the limb." Dr. S. removed the dead protruding bone, (about an inch,) placed the limb in an easy position, and left the patient, deferring the application of splints till next day, in order to meet another surgeon who had been sent for. They were then applied; the wound was cleansed of maggots, and closed as nearly as possible by adhesive strips, and the heel dressed. The patient was discharged in the spring. "His leg has become strong, and he walks without difficulty, and without much lameness." The patient subsequently brought an action for mal-practice against Drs. Goodyear and Hyde, but, after the above facts were elicited upon the trial, the suit was abandoned. Dr. Shipman, attacked in the county press for his share in the affair, published a statement from which we have condensed this account.

This case has elicited the comments of most of the medical journals. In common with the American Journal of the Medical Sciences, the N. Y. Medical Gazette, the Louisville Journal of Medicine and Surgery, we united in condemning the practice of Drs. Goodyear and Hyde in neglecting to replace the protruding bone, and proposing amputation, "on account of the age and intemperate habits of the patient, and the state of the weather, and for fear that a fever might *set in* and carry him off." For his manly and successful efforts to save the limb of this poor fellow, Dr. Shipman deserves great praise, although it seems a clique of his professional brethren in the neighbourhood are weak enough to attempt to put him down for doing his duty. The following proceedings of the Cortland County Medical Society do little credit to that body :—

"At the annual meeting of the Cortland County Medical Society held in Homer Village on the 19th January, it was

Voted, that the following be added to the by-laws of the Society, viz: If any member shall instigate a prosecution against another member of this Society, for mal-practice, before he shall have submitted the same at an annual meeting of the Society and given thirty days notice to the accused of his intention, before said accusation shall be made, and shall have obtained a vote of two-thirds of the members present, declaring the same to be mal-practice, he shall be expelled from this Society.

The following resolution was presented, discussed and laid over to the next meeting of the Society, for the reason that the presiding officer, Dr. A. B. Shipman, refused to put the question when the resolution was moved and seconded, and also refused to leave the chair when requested to do so, when it was well known to the Society that there would have been an almost unanimous vote in favour of the resolution.

Resolved, That on review of the facts in relation to the prosecution by Wm. Smith, against Drs. Goodyear and Hyde, for mal-practice, we have as yet seen nothing to diminish our confidence in their skill as practical Surgeons.

Voted, The proceedings of this meeting be published in the papers of this County, and in the Philadelphia and Boston Medical Journals.

M. Goodyear, Pres't.

*G. W. Bradford*, Sec."

These resolutions, besides endorsing bad surgery, have another obvious ill tendency. They create among the public an impression that physicians are disposed to screen each other from the just consequences of ignorance and incapacity, that they regard their duty to their patients as secondary, and that, as in the present instance, they deem the preservation of limb and life as of little weight in the balance with the observance of a false code of professional etiquette. From principles such as these we feel bound to express our strong dissent. Without entering into the warfare of the Cortland co. physicians, we must say that Dr. Shipman's course, in rescuing his patient from the impending calamity of amputation, appears to have been in accordance with principles of sound surgery and correct ethics.

---

### MISCELLANEOUS INTELLIGENCE.

In the Boston Medical and Surgical Journal of the 26th of January, L. M. Beardsley reports a case of triplets; the children all doing well, two weeks after labor.

In a late number of the same journal, the editor speaks with high encomium of a letter from Dr. Day, of Liberia, stating that he had no previous knowledge of this gentleman, but anticipates advantages to science from his presence in Africa. Dr. Day, graduated at the University of Pennsylvania, from the office of Dr. H. Chase of this city, in 1840. He is a native of New Jersey, of Quaker ancestry, and soon accepted the station of Surgeon in Liberia, from an ardent devotion to the cause of colonization. Since his residence there, he took the temporary editorship of one of the two public papers, during the absence of the regular incumbent, and became engaged in some political discussions which drew on him the ill feeling of some of the enemies of the late lamented Governor Buchanan. We have heard it rumored that he may be induced to return to this country, for this or other reasons; but much as friendship and high respect would lead us to be pleased with his presence, we trust that the sense of duty which has led him already to make so many sacrifices will still longer retain him in a station where the interests of the colony and those of science are likely to be essentially promoted by his exertions. Our brother of the North cannot estimate him too highly. R. C.

